# Influence of host factors and parasite biomass on the severity of imported *Plasmodium falciparum* malaria

**DOI:** 10.1371/journal.pone.0175328

**Published:** 2017-04-14

**Authors:** Nicolas Argy, Eric Kendjo, Claire Augé-Courtoi, Sandrine Cojean, Jérôme Clain, Pascal Houzé, Marc Thellier, Veronique Hubert, Philippe Deloron, Sandrine Houzé, for the CNRP study group

**Affiliations:** 1 Laboratoire de parasitologie, hôpital Bichat-Claude Bernard, APHP, Paris, France; 2 Centre National de Référence du Paludisme, hôpital Bichat-Claude Bernard, APHP, Paris, France; 3 Faculté de Pharmacie, Université Paris Descartes, COMUE Sorbonne Paris Cité, Paris, France; 4 UMR MERIT 216, Institut de recherche pour le développement, Paris, France; 5 Laboratoire de pharmacologie, hôpital Saint-Louis, APHP, Paris, France; 6 Laboratoire de parasitologie, hôpital Pitié-Salpêtrière, APHP, Paris, France; 7 Faculté de Médecine, Université Pierre et Marie Curie, COMUE Sorbonne Paris Cité, Paris, France; Centro de Pesquisas Rene Rachou, BRAZIL

## Abstract

**Objectives:**

Imported malaria in France is characterized by various clinical manifestations observed in a heterogeneous population of patients such as travelers/expatriates and African migrants. In this population, host factors and parasite biomass associated with severe imported malaria are poorly known.

**Methods:**

From data collected by the Centre National de Référence du Paludisme, we identified epidemiological, demographic and biological features including parasite biomass and anti-plasmodial antibody levels (negative, positive and strongly positive serology) associated with different disease severity groups (very severe, moderately severe, and uncomplicated malaria) in 3 epidemiological groups (travelers/expatriates, first- and second-generation migrants).

**Results:**

Age, ethnicity, absence of prior infection with *P*. *falciparum*, antibody levels, plasma PfHRP2 levels, total and circulating parasite biomass were related to severe malaria onset. Sequestered parasite biomass tended to be increased in very severe malaria, and was strongly correlated to the antibody level of the host.

**Conclusions:**

Prior exposure to *P*. *falciparum* is associated with high anti-plasmodial antibody levels which influence clinical presentation of imported malaria and its correlated circulating and sequestered parasite burden.

## Introduction

*Plasmodium falciparum* infection in tropical regions also threatens non-endemic countries through imported cases [1]. Demographic, epidemiological and immunological features of malaria cases diagnosed in Metropolitan France (called imported malaria) are clearly different to those from malaria-endemic areas; the resulting severe forms of malaria are variable [2–5]. Severe malaria (SM) is the consequence of parasitized red blood cell (pRBC) sequestration, responsible for tissue hypoxia and endothelial barrier dysfunction in the microcirculation [6–9], resulting from cytoadherence via host endothelial receptors of extra-erythrocytic parasitic protein *Plasmodium falciparum* Erythrocyte Membrane Protein 1 (PfEMP1) [9–11]. In endemic areas, repeated exposure to *P*. *falciparum* antigens during childhood appears necessary to induce a non-sterilizing antibody-mediated response against antigenic variants of PfEMP1, preventing parasite sequestration in adults [12]. Because of the lack of or waning anti-malarial immunity, travelers and African migrants are at risk of SM [12].

Plasma concentrations of *P*. *falciparum* histidine-rich protein 2 (PfHRP2) have been evaluated as a tool to improve the definition of SM in malaria-endemic areas [13–15]. Plasma PfHRP2 levels, specifically produced by *P*. *falciparum* and released by schizonts, were reported to better reflect the total parasite biomass, including the sequestered component, than peripheral blood parasite density [13, 14].

Various epidemiological risk factors have already been identified in severe imported malaria [16–18]. However, the influences of host characteristics and parasite biomass on the clinical presentation of imported malaria have not yet been investigated. Based on the clinical description of cases [2, 3, 19], we aimed to identify demographic, epidemiological and biological features associated with severe or non-severe imported malaria in France, including evaluation of plasma PfHRP2 levels and parasite biomass.

## Materials and methods

### Ethics statement

No specific consent was required because of, in coordination with Santé Publique France organisation for the care and surveillance of malaria, the human clinical, epidemiological and biological data as well as parasitic biomarkers (parasitaemia, PfHRP2, total parasite biomass, total circulating parasite biomass and sequestered parasite biomass) were collected in the CNRP database and analysed in accordance with the common public health mission of all French National Reference Centers (https://www.legifrance.gouv.fr/affichTexte.do?cidTexte=JORFTEXT000000810056&dateTexte=&categorieLien=id). The study of the biological samples obtained in the medical care context was considered as non-interventional research (article L1221-1.1 of the French public health code) only requiring the non-opposition of the patient during sampling (article L1211-2 of the French public health code). All data collected were anonymized before analyse.

### Data sources and sample collection

This study was conducted in the Centre National de Référence du Paludisme (CNRP) laboratory, between 2010 and 2013. Prospective notification of imported malaria cases was undertaken through the CNRP national network of hospital laboratories. For each case, demographic (age, sex), epidemiological (ethnic origin, visited endemic area, self-reported prior exposure of malaria, immunosuppression, onset of symptoms, onset of diagnosis), biological (platelet counts, arterial blood pH, lactatemia, parasitemia, hemoglobin, creatinine, blood glucose, bilirubinemia) and clinical data (neurological and coagulation disorders, respiratory and renal failure, shock and jaundice) were collected in a database by the corresponding hospital, and the blood sample used for the initial parasitological diagnosis (D0 sample) was forwarded to the CNRP laboratory for diagnostic evaluation. Among these cases, inclusion criteria were as follows: patients not living in malaria-endemic countries and Caucasian expatriates infected with *P*. *falciparum*, D0 whole blood sample available in sufficient quantity to perform planned laboratory assays, no curative or preventive malaria treatment in the 30 days before diagnosis as confirmed by plasma determination of antimalarial drugs and metabolites at D0 (monodesethylchloroquine, chloroquine, cycloguanil, proguanil, quinine, doxycycline, sulfadoxine, pyrimethamine, mefloquine, lumefantrine, desbutyl-lumefantrine, amodiaquine, desethylamodiaquine, carboxymefloquine, atovaquone, piperaquine, primaquine and desbutylhalofantrine) and absence of bacterial co infection confirmed by negative blood culture during hospitalization.

### Laboratory procedures

*Plasmodium* species and parasite density were determined from D0 samples on stained blood films. Parasitemia was expressed as number of parasites per μL assuming a mean of 4,500,000 RBC/μL. In the event of low parasite density, DNA extraction and nested species-specific PCR [20, 21] were performed to confirm malaria diagnosis. D0 plasma samples were stored at -20°C and used for antimalarial drug testing, PfHRP2 assay and serologic testing.

### Study population

Patients were classified into 3 mutually exclusive groups: first-generation migrants (FGM) (born in malaria-endemic regions and living in Metropolitan France), second–generation migrants (SGM) (born in Metropolitan France from FGM parents and living in Metropolitan France), and travelers/expatriates (T/E). Travelers were born and living in non-malaria-endemic regions whereas expatriates were born in non-malaria-endemic regions and living in malaria-endemic regions. Malaria cases were classified into 2 groups: severe malaria (SM) defined according to the WHO definition [22], and uncomplicated malaria (UM) defined by the presence of asexual parasites and fever without any severity criteria. Following French recommendations regarding the classification of imported malaria cases [19], SM cases were also split into two sub-groups: very severe malaria (VSM), associated with a poor prognosis in the imported malaria context [19], defined by the presence of either organ dysfunction or shock or hyperlactatemia (or a combination of those) and moderately severe malaria (MSM), associated with a better prognosis [19], defined by the presence of one or several of the other WHO severity criteria.

### PfHRP2 enzyme-linked immunosorbent assay and estimation of total parasite biomass

PfHRP2 assays were performed with the Malaria Antigen Celisa^®^ commercial kit (Cellabs^®^; Brookvale, Australia), as recommended by the manufacturer, on D0 plasma diluted in RPMI 1640 (Life technologies^®^, Carlsbad, California, USA), according to the initial parasite density. PfHRP2 was quantified with the calibration curve obtained from three dilutions of the positive control. Samples with optical density values outside the range of the calibration were retested at an adapted dilution.

Total parasite biomass was estimated from the plasma PfHRP2 value, as described by Hendriksen et al. [15]. Total parasite biomass (Ptot) (number of parasites) was estimated by the formula: Ptot = 7.3 x PfHRP2 (g/L) x (1-hematocrit (%)) x body weight (kg) x 10^13^. The estimated total circulating parasite biomass (Pcirc) (number of parasites) was calculated as follows: Pcirc = parasites/μL x 10^6^ x blood volume (L). The blood volume was defined as 0.08 x body weight (kg). The estimated sequestered parasite biomass (Pseq) corresponded to the difference between the estimated total parasite biomass and the estimated total circulating parasite biomass such as Pseq (number of parasites) = Ptot (number of parasites)–Pcirc (number of parasites) [15]. We assumed, as described in [15], that patients with negative Pseq values had few or no sequestered parasites, and these negative values were considered 0.

### Indirect immunofluorescence assay

Previous exposure to *Plasmodium sp*. was evaluated by the detection and the quantification of total antibodies against *Plasmodium falciparum*. IgG/A/M anti-plasmodial antibodies were detected and quantified by serological screening based on indirect immunofluorescence assay using whole schizonts of the 3D7 *P*. *falciparum* strain as crude antigens, and fluoresceine linked anti-human IgG/A/M (Biorad^®^; Hercules, California, USA) as conjugate. Quantification of plasmatic antibody concentration was estimated by serologic titers. A four-fold dilution series of plasma from 1:16 to 1:16,384 were performed to determine the titer of anti-plasmodial antibodies corresponding to the final dilution for which fluorescent signal remains positive. Slides were examined under blinded conditions by two experienced microscopists. In case of discordant result, a third microscopist observed in a blinded manner the slide to arbitrate. The validation of the method determined the threshold of positivity at a titer of (1:64) corresponding to the threshold with the best compromise between sensitivity and specificity for antibody detection. Antibody titers from (1:64) to (1:1024) are usually observed at malaria remission. Titers over or equal to (1:4096) are unusual. For statistical analysis, antibody titers were classified into three groups: negative (or 0) corresponding to titers < 1:64; positive, corresponding to titers 1:64, 1:256 and 1:1024 and strongly positive, corresponding to titers ≥ 1:4096.

### Statistical analysis

For each SM case included, control patients were randomly chosen from the pool of patients with UM. Quantitative variables were represented by mean and standard deviation (SD) for normal distributions, and by median and interquartile range [25th percentile-75th percentile] for non-normal distributions. Categorical variables were evaluated according to size and frequency. Quantitative variables with highly skewed distributions (plasma PfHRP2 concentration and parasite biomass values (Ptot, Pcirc and Pseq)) were log-transformed for statistical analysis and graphic representation. Chi-square test and Fisher’s exact test were used to compare categorical variables between groups. ANOVA and Kruskall-Wallis tests were used to compare quantitative variables between groups when appropriated. For all tests, a difference was considered significant when p<0.05. All reported p values are two-tailed. Bonferroni’s correction was performed for multiple comparisons between quantitative variables and groups.

The association between the presence of anti-plasmodial antibody as binary variable (positive/negative serology) and adjusted potential risk factors (theoretical and/or significant) as disease severity sub-group (VSM, MSM, UM), epidemiological sub-group (FGM, SGM and T/E) and age as binary variable (age<18 years-old/age>18 years-old) was investigated using logistic regression. Secondly, the association between imported malaria onset and adjusted potential risk factors (theoretical and/or significant) as the categorical variable epidemiological sub-groups (FGM, SGM and T/E) and antibody levels as well as age, total, circulating and sequestered parasite biomass as continuous variable was also investigated by logistic regression. Four multiple linear regressions models were finally performed to identify influential factors of PfHRP2 and parasite biomass (Ptot, Pcirc and Pseq) as the continuous variable age, disease severity and epidemiological sub-groups and antibody levels. Significant positive or negative relationships between variables were expressed by the coefficient values. For regression models, two-way interaction terms between variables were tested, but none were significant. Statistical analyses were performed using STATA, version 12 (Stata corp^®^, College station, Texas, USA).

## Results

### Study population

Among the 333 *P*. *falciparum* imported malaria cases included in the study, 138 were severe malaria cases (41.4%) comprising 55 very severe malaria and 83 moderately severe malaria cases, and 195 were uncomplicated malaria cases (58.6%). Demographic, epidemiological, clinical and biological characteristics of the UM group and SM sub-groups are presented in Table 1. 215 patients were men (64.6%) and the mean age was 37.3±16.3 years. 215 patients were first-generation migrants (64.6%), 52 second-generation migrants (15.6%), and 66 travellers/expatriates (19.8%). Patients were infected in West Africa (68.5%), Central Africa (26.4%), East Africa (1.5%) and the Indian Ocean (3%).

**Table 1:** Demographic, epidemiological, clinical and biological data from imported malaria cases in Metropolitan France from 2010 to 2013.

	SM (n = 138)		UM (n = 195)	P value*
	VSM (n = 55)	MSM (n = 83)		
Demographic data				
Age (years)†	44.4 ±17	36.3 ±14.8	35.7 ±16.3	0.002^a^
Men	36 (16.7)	51 (23.7)	128 (59.6)	0.8
Women	19 (16.1)	32 (27.1)	67 (56.8)	
Epidemiological data				
1^st^ generation migrant	31 (14.4)	50 (23.2)	134 (62.3)	0.002
2^nd^ generation migrant	5 (9.7)	11 (21.1)	36 (69.2)	
Travelers/expatriates	19 (28.8)	22 (33.3)	25 (37.9)	
Country of infection				
West Africa	32 (14)	53 (23.2)	143 (62.8)	0.09
Central Africa	18 (20.4)	24 (27.3)	46 (52.3)	
East Africa	1 (20)	1 (20)	3 (60)	
Indian Ocean	3 (30)	4 (40)	3 (30)	
Caribbean	0 (0)	1 (100)	0 (0)	
Asia	1 (100)	0 (0)	0 (0)	
History of malaria§	8 (11.3)	13 (18.3)	50 (70.4)	0.03
Immunosuppression¶	6 (30)	7 (35)	7 (35)	0.06
Onset of symptoms (days)‡^♯^	3 [0–8]	3 [0–8]	3 [0–9]	0.28^b^
Onset of diagnosis (days)‡ ^&^	4 [2–6]	3 [2–5]	3 [2–4]	0.04^b^
Clinical data				
Neurological disorders^+^	30 (55)	0 (0)	0 (0)	NA
Coma	1 (2)	0 (0)	0 (0)	
Shock	17 (31)	0 (0)	0 (0)	
Respiratory failure	7 (13)	0 (0)	0 (0)	
Hemorrhagic syndrome	1 (2)	1 (1)	0 (0)	
Hemoglobinuria	4 (7)	7 (9)	0 (0)	
Jaundice	19 (35)	12 (15)	0 (0)	
Biological data				
Platelets (Giga/L)‡	37 [21–69]	66 [42–108]	96 [64–142]	0.0001^b^
Parasitemia (10^3^*parasites/μL)‡	360 [135–720]	270 [180–450]	45 [18–72]	0.0001^b^
Acidosis (pH<7.35)	7 (13)	0 (0)	0 (0)	NA
Lactacidemia (>2.2 mmol/L)	27 (31)	0 (0)	0 (0)	
Hyperparasitemia (>180.000 parasites/μL)	40 (73)	65 (79)	0 (0)	
Anemia (Hb<8 g/dL)	3 (5)	4 (5)	0 (0)	
Creatinine (>265 μmol/L)	15 (27)	0 (0)	0 (0)	
Blood glucose (<2.2 mmol/L)	2 (4)	0 (0)	0 (0)	
Bilirubinemia (>50μmol/L)	24 (44)	14 (58)	0 (0)	

SM, severe malaria; VSM, very severe malaria; MSM, moderately severe malaria; UM, uncomplicated malaria; Hb, hemoglobin; NA, not applicable.

Categorical data are presented as absolute numbers (%).

* Chi-square test and Fisher’s exact test were used to compare categorical variables between groups. Significant difference when p<0.05.

^†^Normally distributed variables are represented by their mean ± standard deviation.

^a^ ANOVA was used to compare parametrics quantitative variables between groups. Significant difference when p<0.05.

^§^ Existence of a prior self-reported malaria infection.

^¶^ Immunosuppressive status includes HIV infection (95%) and iatrogenic immunosuppression (5%).

^‡^ Non-normally distributed data are shown as median [25^th^ percentile-75^th^ percentile].

^b^ Kruskall-Wallis tests was used to compare non-parametrics quantitative variables between groups. Significant difference when p<0.05.

^♯^ The onset of symptoms is the number of days between the return from endemic regions and the onset of clinical symptoms.

^&^ The onset of diagnosis is the number of days between the onset of symptoms and the diagnosis in hospital. In our cohort, treatment started on the day of diagnosis.

^+^ Neurological disorders included obnubilation, confusion, drowsiness and prostration [19].

Age of patients varied according to disease severity sub-groups (p = 0.002) (Table 1), with very severe malaria patients being older than moderately severe malaria and uncomplicated malaria patients (p = 0.013 and p = 0.001 respectively). The proportions of first-generation migrants, second-generation migrants and travelers/expatriates differed according to malaria severity (p = 0.002) (Table 1), the highest proportion of first-generation migrants and second-generation migrants was observed in the uncomplicated malaria group whereas travelers/expatriates were more common in the very severe malaria and moderately severe malaria groups. Previous self-reported history of malaria was more frequent in uncomplicated malaria than in very severe malaria and moderately severe malaria groups (p = 0.03) (Table 1). The median time between onset of symptoms and diagnosis differed between clinical sub-groups (p = 0.04), being higher in the very severe malaria group (4 days [2–6]) (Table 1). There was a gradual significant decrease in platelets related to disease severity decrease, from uncomplicated malaria to very severe malaria groups (p<0.01). Median parasite density varied according to disease severity (p<0.001) with parasite density being higher in severe malaria than in uncomplicated malaria (p<0.01), but being similar in very severe malaria and moderately severe malaria groups.

### PfHRP2 and parasite biomass determination

**Fig 1:**
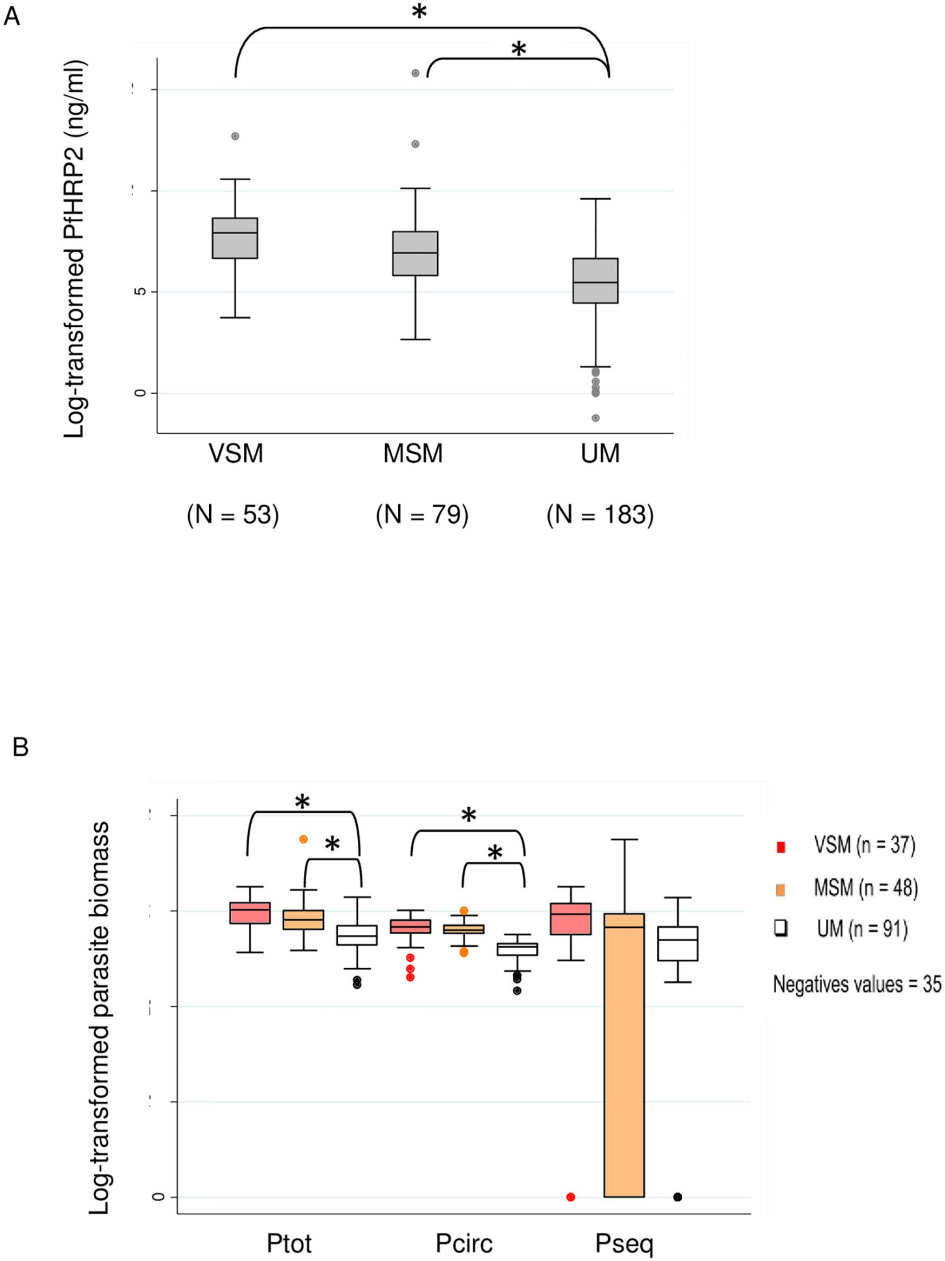
PfHRP2 plasma levels and parasite biomass according to clinical presentation of imported malaria. (A) Log-transformed plasma levels of PfHRP2 in the very severe malaria (VSM), moderately severe malaria (MSM) and uncomplicated malaria (UM) sub-groups. Levels of PfHRP2 were determined on 315 D0 plasma samples by ELISA. (*) corresponds to a significant difference using ANOVA test (p<0.05) and Bonferroni’s correction (p<0.016). (B) Log-transformed estimated total parasite biomass (Ptot), estimated total circulating parasite biomass (Pcirc) and estimated sequestrated parasite biomass (Pseq) respectively in the very severe malaria (VSM) (red box plot), moderately severe malaria (MSM) (orange box plot) and uncomplicated malaria (UM) groups (white box plot). Parasite biomass was estimated for 176 patients using a mathematical approach as described [15] with quantitative plasma levels of PfHRP2 (g/L) determined by ELISA, hematocrit (%), patient body weight (kg) and parasitemia (parasites/μL) on D0 sample. Box plot represents the median [25th percentile-75th percentile] with the extreme value 10th and 90th percentile. (*) corresponds to a significant difference using ANOVA test (p<0.05) and Bonferroni’s correction (p<0.016).

To investigate parasite factors involved in severe malaria onset, plasma concentrations of PfHRP2 and total, circulating and sequestered parasite biomass were determined. Quantification of PfHRP2 was performed for 315 patients including 132 severe malaria cases (53 very severe malaria and 79 moderately severe malaria cases) and 183 uncomplicated malaria cases. The plasma levels of PfHRP2 were significantly higher in the very severe malaria and moderately severe malaria group compared to uncomplicated malaria (2766.9 ng/mL [772–5742.2] and 1017 ng/mL [336.4–2908.9] respectively vs. 234.7 ng/mL [85–774.2]; p<0.001), but were similar in very severe malaria and moderately severe malaria groups (Fig 1A). Total (Ptot), circulating (Pcirc), and sequestered (Pseq) parasite biomass were estimated for the 176 patients for whom weight and hematocrit data were available, including 85 severe malaria cases (37 very severe malaria and 48 moderately severe malaria) and 91 uncomplicated malaria patients. Negative Pseq values were obtained in 35 patients (19.9%) and were reported as 0 [14, 15] (Fig 1B). Total and circulating parasite biomass varied according to disease severity. Significantly higher levels of both Ptot and Pcirc were observed in very severe malaria compared to uncomplicated malaria and in moderately severe malaria compared to uncomplicated malaria (Ptot: 1.2 X 10^13^ [2.8 X 1012–2.5 X 10^13^] in very severe malaria, 4.4 X 10^12^ [1.5 X 10^12^–1.1 X 10^13^] in moderately severe malaria and 7.9 X 10^11^ [3 X 10^11^–2.3 X 10^12^] in uncomplicated malaria, p<0.001 for both comparisons; Pcirc: 2 X 10^12^ [1 X 10^12^–4.2 X 10^12^] in very severe malaria, 1.5 X 10^12^ [9.9 X 10^11^–2.3 X 10^12^] in moderately severe malaria and 2.5 X 10^11^ [1.0 X 10^11^–3.5 X 10^11^] in uncomplicated malaria, p<0.001 for both comparisons), but were similar in very severe malaria and moderately severe malaria groups (Fig 1B). Unexpectedly, Pseq levels did not differ between disease severity sub-groups.

### Anti-plasmodial antibody in plasma

Anti-plasmodial antibody levels in plasma were investigated as host factors potentially influencing clinical presentation of imported malaria. The variation in antibody levels was evaluated according to disease severity and epidemiological groups. The titers of total anti-*P*. *falciparum* plasma antibodies at D0 were determined for 329 patients, and classified as negative in 93 cases (28.3%), positive in 179 cases (54.4%), and strongly positive in 57 cases (17.3%). Antibody levels differed according to disease severity sub-groups (p = 0.023), negative antibody levels were more frequent in moderately severe malaria compared to uncomplicated malaria (41.5% vs. 22.9%; p = 0.003). Antibody levels also varied with regard to malaria exposure (p<0.001). Negative serology was less frequent in the first-generation migrants group (p<0.001) (Table 2).

**Table 2:** Repartition of antibody titer sub-groups in each epidemiological sub-group.

	FGM	SGM	T/E	P value*
**Antibody titer**	No. (%)	No. (%)	No. (%)	
Negative	39 (41.9)	23 (24.7)	31 (33.3)	< 0.001
Positive	125 (69.8)	26 (14.5)	28 (15.6)	
Strongly positive	47 (82.4)	3 (5,3)	7 (12.3)	

FGM, first-generation migrants; SGM, second-generation migrants; T/E, Travelers/expatriates.

Patients with negative (<64), positive (between 64 and 1,024) and strongly positive antibody titers (superior or equal to 4,096), as determined by indirect immunofluorescence assay, are represented in percent (%) for each epidemiological sub-group.

(*) χ^2^ and Fischer’s exact test when appropriate were used to compare categorical variables between epidemiological sub-groups. Significant difference was considered when p<0.05.

In multivariate analysis, positive antibody titers were more likely observed in uncomplicated malaria (odds ratio [95% CI]: 2.2 [1.2–4]; p = 0.008). Negative antibody levels were more likely observed in second-generation migrants and travelers/expatriates groups (0.3 [0.1–0.6]; p = 0.002 and 0.3 [0.2–0.5]; p<0.001 respectively) (Table 3).

**Table 3:** Multivariate analysis with logistic regression to identify factors influencing positive serology at D0 in French imported malaria.

Positive serology	Variable	Odds Ratio	[95% CI]	P value
	Age<18 years	1.00 (reference group)		
	Age>18 years	0.8	[0.3–2.2]	0.7
	MSM	1.00 (reference group)		
	UM	2.2	[1.2–4]	0.008
	VSM	2.1	[1–4.6]	0.06
	FGM	1.00 (reference group)		
	SGM	0.3	[0.1–0.6]	0.002
	T/E	0.3	[0.2–0.5]	<0.001

VSM, very severe malaria; MSM, moderately severe malaria; UM, uncomplicated malaria; FGM, first-generation migrants; SGM, second-generation migrants; T/E, travelers/expatriates; CI, confidence interval.

Multivariate analysis was performed on 329 patients and age (as a binary variable: <18 years-old or >18 years-old), clinical sub-group (VSM, MSM, UM) and epidemiological sub-groups (FGM, SGM and T/E) were selected, as theoretical influent factors (age) or after univariate analysis (clinical and epidemiological sub-groups). Age< 18 years old, MSM and FGM were chosen as reference groups. Factors were considered to have a significant influence if p value<0.05.

### Risk factors for clinical severity

Logistic regression was performed to identify risk factors for severe imported malaria onset from age, ethnic origin, antibody levels, and parasite biomass (S1–S4 Tables). This multivariate analysis identified, PfHRP2, Ptot and Pcirc as risk factors of MSM (odds ratio [95% CI]: 1.3 [1.2–1.6]; 1.3 [1.1–1.6], and 2.7 [1.9–3.9] respectively; all p<0.01) (S3 Table), and of VSM (1.6 [1.3–2.0]; 1.5 [1.2–1.9], and 2.1 [1.5–2.9], respectively; all p<0.01) (S2 Table). Conversely, decreased PfHRP2, Ptot and Pcirc were associated to uncomplicated malaria (0.5 [0.5–0.6]; 0.5 [0.4–0.7] and 0.1 [0.1–0.3] respectively; all p<0.01) (S4 Table). Depending on the statistical model, age was also a significant influent factor of very severe and moderately severe malaria onset (1 [1–1.1]; p = 0.01 and 1 [0.2–1]; p = 0.006 respectively) (S2 and S3 Tables) as well as positive serology sub-groups for moderately severe malaria and uncomplicated malaria (0.2 [0.1–0.7]; p = 0.01 and 4.4 [1.2–16.6]; p = 0.03 respectively) (S3 and S4 Tables).

Linear regression was performed to identify variables influencing PfHRP2 and parasite biomass values considered as risk factors of severity, from significant variables previously identified between disease severity sub-groups (VSM, MSM and UM) and from theoretical variables (age, epidemiological and antibody titer sub-groups) (Table 4). A negative correlation was observed between the UM group and PfHRP2, Ptot or Pcirc (coefficient [95% CI]: -1 [-2.1 - -1.2], -1.7 [-2.4 - -1.1] and -2.1 [-2.5 - -1.7]; all p<0.001), between negative antibody levels and PfHRP2 (-0.96 [-1.6 - -0.3]; p = 0.004) or Ptot (-1.1 [-1.2 - -0.3]; p = 0.009). Positive correlation was observed between positive antibody levels and Pcirc (0.5 [0.001–0.98]; p = 0.049). Pseq was positively correlated with the VSM group (6.5 [1.6–11.5]; p = 0.01) and was negatively correlated with negative and positive antibody titer groups (-7.8 [-13.2 - -2.3] and -6.7 [-11.6 - -1.7]; both p<0.01) (Table 4).

**Table 4:** Models of multivariate analysis with linear regression to identify factors related to PfHRP2, estimated total parasite biomass, estimated total circulating parasite biomass and estimated sequestered parasite biomass in imported malaria in France, 2010–2013.

Model		Risk factors	Coefficient (standard error)	[95% CI]	P value
Model 1: PfHRP2		Intercept	7 (0.44)	[6.1–7.9]	< 0.0001
		Age	0.0007 (0.008)	[-0.01–0.01]	0.93
	Disease severity sub-groups				
		VSM	0.6 (0.3)	[-0.08–1.2]	0.09
		UM	-1.6 (0.25)	[-2.1- -1.2]	<0.001
	Epidemiological sub-groups				
		SGM	0.4 (0.3)	[-0.3–1.1]	0.22
		T/E	0.5 (0.3)	[-0.01–1.1]	0.06
	Antibody titer sub-groups				
		Positive	-0.3 (0.3)	[-0.9–0.2]	0.23
		Negative	-0.96 (0.3)	[-1.6- -0.3]	0.004
Model 2: Ptot		Intercept	28.5 (0.4)	[27.6–29.3]	< 0.0001
		Age	0.002 (0.01)	[-0.02–0.02]	0.83
	Disease severity sub-groups				
		VSM	0.5 (0.4)	[-0.3–1.2]	0.23
		UM	-1.7 (0.3)	[-2.4- -1.1]	<0.001
	Epidemiological sub-groups				
		SGM	-0.3 (0.4)	[-1.2–0.6]	0.52
		T/E	0.3 (0.4)	[-0.5–1.0]	0.48
	Antibody titer sub-groups				
		Positive	-0.7 (0.4)	[-1.5–0.07]	0.08
		Negative	-1.1 (0.4)	[-2.0- -0.3]	0.009
Model 3: Pcirc		Intercept	26.5 (0.3)	[25.9–27.1]	< 0.0001
		Age	0.02 (0.01)	[0.004–0.03]	0.01
	Disease severity sub-groups				
		VSM	-0.1 (0.2)	[-0.6–0.3]	0.55
		UM	-2.1 (0.2)	[-2.5- -1.7]	<0.001
	Epidemiological sub-groups				
		SGM	-0.3 (0.3)	[-0.9–0.22]	0.23
		T/E	0.1 (0.2)	[-0.3–0.6]	0.61
	Antibody titer sub-groups				
		Positive	0.5 (0.2)	[0.001–0.98]	0.049
		Negative	0.5 (0.3)	[-0.006–1.1]	0.052
Model 4: Pseq		Intercept	26.5 (2.9)	[21–32.2]	< 0.0001
		Age	-0.08 (0.07)	[-0.2–0.05]	0.23
	Disease severity sub-groups				
		VSM	6.5 (2.5)	[1.6–11.5]	0.01
		UM	1.2 (2.1)	[-2.9–5.2]	0.6
	Epidemiological sub-groups				
		SGM	-0.4 (2.9)	[-6.0–5.3]	0.9
		T/E	1.9 (2.4)	[-2.8–6.6]	0.42
	Antibody titer sub-groups				
		Positive	-6.7 (2.5)	[-11.6- -1.7]	0.009
		Negative	-7.8 (2.8)	[-13.2- -2.3]	0.005

VSM, very severe malaria; MSM, moderately severe malaria; UM, uncomplicated malaria; FGM, first-generation migrants; SGM, second-generation migrants; T/E, travelers/expatriates; CI, confidence interval.

A linear regression was performed on 315 patients for PfHRP2 and on 176 patients for Ptot, Pcirc and Pseq. Age, disease severity sub-group (VSM, MSM, UM), epidemiological sub-group (FGM, SGM, T/E) and antibody titer sub-groups (negative, positive, strongly positive) were selected as potential factors after univariate analysis. MSM, FGM and strongly positive antibody titer sub-groups were chosen as reference. PfHRP2, Ptot, Pcirc, Pseq were log-transformed for statistical analysis. Significance was set to p<0.05.

## Discussion

A wide variety of clinical presentation could be seen in the context of imported malaria. Through the CNRP network and database, the study of imported severe and non-severe malaria cases, as defined by the WHO criteria established in 2000 and still in force in France, reaffirmed since 2013 [23] despite the 2014 WHO criteria [24], allowed us to identify risk factors in susceptible populations.

As reported previously, severe malaria patients, and particularly very severe malaria patients, are older than uncomplicated malaria patients [2, 16, 25], and differ in ethnicity [3, 16, 18], delay between onset of symptoms and diagnosis [18], previous history of malaria [16] and thrombocytopenia [26]. High parasite density is more frequent in SM, but similar in very severe malaria and moderately severe malaria patients. Among epidemiological sub-groups with a risk factor in severe malaria, travelers/expatriates are most susceptible to very severe malaria. Multivariate analysis including all these significant variables confirmed a significant association between age, serological status at D0 and severe imported malaria onset. Serological status is theoretically correlated with pre exposure to malaria and also associated with epidemiological sub-groups (second-generation migrants and travelers/expatriates) and disease severity sub-groups (UM) as observed in multivariate analysis. Compared to 70.4% in the uncomplicated malaria group which reported prior history of malaria, absence of or low prior exposure to *P*. *falciparum* in travelers/expatriates may explain this difference in clinical presentation. The high rate of prior history of malaria reported in 73.2% in the first-generation migrants groups corroborated our observations. Accordingly, it may be hypothesized that the potential protective effect of prior exposure to *P*. *falciparum* against SM, likely depends on the frequency and number of prior exposures [12, 27, 28]. Antibody detection and titers are informative with regard to prior exposure to *Plasmodium spp*. but not with regard to anti-malarial immune protection, providing quantitative but not qualitative information on the antibody response. The hypothesis of probable long-term protection in first-generation migrants is consistent with our results [29]. Persistence of protection after leaving malaria-endemic areas has already been suggested, but few biological data on immune responses are available [29, 30]. However, the differences in clinical presentation, observed in second-generation migrants and travelers/expatriates suggest the possibility that other factors are involved in protection against SM [31, 32]. Moreover, the difference in antibody response between first-generation migrants and second-generation migrants may also result from differences in exposure to the parasite.

Plasma PfHRP2 concentrations, Ptot and Pcirc correlated with the severity of malaria infection as observed in univariate and multivariate statistical analysis, but did not differentiate severe forms [14, 33]. Mathematical modeling to calculate parasite biomass may require taking into account both the *P*. *falciparum* strain and the population studied, which are thus central factors in this limitation, as already discussed by Dondorp et al., [14]. However, the lack of power or other pathophysiological mechanisms may also impede the detection of differences between SM sub-groups [14]. As we used the same methodology to estimate parasite biomass, this bias likely also applies to our work. As described by Cunnington et al., [34] sequestered parasite biomass is similar between SM and UM, confirming that parasite sequestration also occurs in UM. Distinct adhesion phenotypes may explain differences in malaria pathogenesis and in clinical presentation [11]. High antibody levels probably limit sequestration in UM, or favor an adhesion phenotype switch. However, linear regression model revealed a strong interaction between sequestered parasite biomass and VSM confirming a key role of the sequestration in very severe malaria. As observed for PfHRP2, Ptot and Pcirc, significant correlation between Pseq and the host antibody response was also observed, revealing interaction between parasite biomass and antibodies response.

Our study utilizing the CNRP network is subject to limitations. Recording of imported malaria cases is estimated to be 52% complete, and the percentage of sample transfer after notification was 75.8% in 2014, leading to a possible underestimation of particular clinical presentations as cerebral malaria [35]. Moreover, inclusion criteria with negative drug plasma levels to avoid the influence of drugs on biological data have limited the number of patients included, as antimalarial self-medication was frequently detected. Non-exhaustive information is sufficient for notification of imported malaria cases to the CNRP database, meaning that specific data may be missing, eventually preventing calculation of the parasite biomass. Determination of PfHRP2 was not performed if sample volume was inadequate. Pediatric cases were not included in our study because of insufficient blood sample volume, leading to skewed recruitment of children.

Our study of imported malaria confirms that ethnic origin, and anti-*P*. *falciparum* antibody response, correlated to prior exposure to *P*. *falciparum*, influent the clinical presentation of imported malaria. Parasite biomass may also determine the nature of the clinical presentation and significantly interact with antibodies titers. Our data suggest the key role of prior exposure to *P*. *falciparum* in inducing protection against VSM, generating antibody levels which significantly reduce the circulating and sequestered parasite burden. A more accurate qualitative study of the protective antibody repertoire is needed to understand the host-parasite interactions in non-exposed patients in imported malaria context.

## Supporting information

S1 Table Correlation matrix between plasmatic HRP2, estimated total parasite biomass, estimated total circulating parasite biomass and estimated sequestered parasite biomass. Ptot, estimated total parasite biomass; Pcirc, estimated total circulating parasite biomass; Pseq, estimated sequestered parasite biomass.
(DOCX)

S2 Table Models of multivariate analysis with logistic regression to identify factors influencing very severe malaria onset in French imported malaria. FGM, first-generation of migrants; SGM, Second-generation of migrants; T/E, Travellers/expatriates; VSM, very severe malaria; CI, Confidence interval; Ptot, estimated total parasite biomass; Pcirc, estimated total circulating biomass; Pseq, estimated sequestered parasite biomass. Multivariate analysis was performed on 315 patients for PfHRP2 (model 1) and 176 patients for Ptot, Pcirc and Pseq (model 2–4 respectively). Age, malaria exposure, serology status and log-transformed PfHRP2, Ptot, Pcirc and Pseq were selected, considered as influent factors. FGM and strongly positive serology were chosen as reference group for the analysis whereas age, PfHRP2, Ptot, Pcirc and Pseq were considered as continuous variable. Factors have a significant influence if p value<0.05.
(DOCX)

S3 Table Models of multivariate analysis with logistic regression to identify factors influencing moderately severe malaria onset in French imported malaria. FGM, first-generation of migrants; SGM, Second-generation of migrants; T/E, Travellers/expatriates; VSM, very severe malaria; CI, Confidence interval; Ptot, estimated total parasite biomass; Pcirc, estimated total circulating biomass; Pseq, estimated sequestered parasite biomass. Multivariate analysis was performed on 315 patients for PfHRP2 (model 1) and 176 patients for Ptot, Pcirc and Pseq (model 2–4 respectively). Age, malaria exposure, serology status and log-transformed PfHRP2, Ptot, Pcirc and Pseq were selected, considered as influent factors. FGM and strongly positive serology were chosen as reference group for the analysis whereas age, PfHRP2, Ptot, Pcirc and Pseq were considered as continuous variable. Factors have a significant influence if p value<0.05.
(DOCX)

S4 Table Models of multivariate analysis with logistic regression to identify factors influencing uncomplicated malaria onset in French imported malaria. FGM, first-generation of migrants; SGM, Second-generation of migrants; T/E, Travellers/expatriates; VSM, very severe malaria; CI, Confidence interval; Ptot, estimated total parasite biomass; Pcirc, estimated total circulating biomass; Pseq, estimated sequestered parasite biomass. Multivariate analysis was performed on 315 patients for PfHRP2 (model 1) and 176 patients for Ptot, Pcirc and Pseq (model 2–4 respectively). Age, malaria exposure, serology status and log-transformed PfHRP2, Ptot, Pcirc and Pseq were selected, considered as influent factors. FGM and strongly positive serology were chosen as reference group for the analysis whereas age, PfHRP2, Ptot, Pcirc and Pseq were considered as continuous variable. Factors have a significant influence if p value<0.05.
(DOCX)
