# Supplementary material for: Influence of host factors and parasite biomass on the severity of imported Plasmodium falciparum malaria
Source: PLoS One. 2017 Apr 14;12(4):e0175328. doi: 10.1371/journal.pone.0175328 (PMC5391917; doi:10.1371/journal.pone.0175328)
Supplement: S1 Table — Ptot, estimated total parasite biomass; Pcirc, estimated total circulating parasite biomass; Pseq, estimated sequestered parasite biomass. (DOCX) [file pone.0175328.s001.docx]

**S1 Table: Correlation matrix between plasmatic HRP2, estimated total parasite biomass, estimated total circulating parasite biomass and estimated sequestered parasite biomass.**

|  | PfHRP2 | Ptot | Pcirc | Pseq |
| --- | --- | --- | --- | --- |
| PfHRP2 | 1 |  |  |  |
| Ptot | 0.97 | 1 |  |  |
| Pcirc | 0.44 | 0.5 | 1 |  |
| Pseq | 0.64 | 0.63 | 0.02 | 1 |

Ptot, estimated total parasite biomass; Pcirc, estimated total circulating parasite biomass; Pseq, estimated sequestered parasite biomass.
