# Supplementary material for: Influence of host factors and parasite biomass on the severity of imported Plasmodium falciparum malaria
Source: PLoS One. 2017 Apr 14;12(4):e0175328. doi: 10.1371/journal.pone.0175328 (PMC5391917; doi:10.1371/journal.pone.0175328)
Supplement: S2 Table — FGM, first-generation of migrants; SGM, Second-generation of migrants; T/E, Travellers/expatriates; VSM, very severe malaria; CI, Confidence interval; Ptot, estimated total parasite biomass; Pcirc, estimated total circulating biomass; Pseq, estimated sequestered parasite biomass. Multivariate analysis was performed on 315 patients for PfHRP2 (model 1) and 176 patients for Ptot, Pcirc and Pseq (model 2–4 respectively). Age, malaria exposure, serology status and log-transformed PfHRP2, Ptot, Pcirc and Pseq were selected, considered as influent factors. FGM and strongly positive serology were chosen as reference group for the analysis whereas age, PfHRP2, Ptot, Pcirc and Pseq were considered as continuous variable. Factors have a significant influence if p value<0.05. (DOCX) [file pone.0175328.s002.docx]

**S2** **Table: Models of multivariate analysis with logistic regression to identify factors influencing very severe malaria onset in French imported malaria.**

| Model | Variable | OR (standard error) | [95% CI] | P value |
| --- | --- | --- | --- | --- |
| Model 1 : VSM | age | 1 (0.01) | [1-1.1] | 0.01 |
|  | FGM | 1.00 (reference group) |  |  |
|  | SGM | 1.3 (0.82) | [0.4-4.8] | 0.7 |
|  | T/E | 1.5 (0.57) | [0.7-3.2] | 0.3 |
|  | Strongly positive serology | 1.00 (reference group) |  |  |
|  | Negative serology | 1.2 (0.6) | [0.4-3.2] | 0.8 |
|  | Positive serology | 1.2 (0.54) | [0.5-2.9] | 0.7 |
|  | PfHRP2 | 1.6 (0.16) | [1.3-2.0] | <0.001 |
| Model 2 : VSM | age | 1 (0.02) | [1-1.1] | 0.1 |
|  | FGM | 1.00 (reference group) |  |  |
|  | SGM | 1.1 (0.86) | [0.2-5] | 0.7 |
|  | T/E | 1.7 (0.83) | [0.6-4.4] | 0.3 |
|  | Strongly positive serology | 1.00 (reference group) |  |  |
|  | Negative serology | 1.5 (1.0) | [0.4-5.5] | 0.5 |
|  | Positive serology | 1.6 (0.9) | [0.5-5] | 0.4 |
|  | Ptot | 1.5 (0.18) | [1.2-1.9] | <0.001 |
| Model 3: VSM | age | 1 (0.02) | [1-1.1] | 0.2 |
|  | FGM | 1.00 (reference group) |  |  |
|  | SGM | 1.7 (1.3) | [0.4-7.7] | 0.5 |
|  | T/E | 1.8 (0.9) | [0.7-4.7] | 0.2 |
|  | Strongly positive serology | 1.00 (reference group) |  |  |
|  | Negative serology | 0.6 (0.4) | [0.2-2.3] | 0.5 |
|  | Positive serology | 0.9 (0.5) | [0.3-2.9] | 0.9 |
|  | Pcirc | 2.1 (0.4) | [1.5-2.9] | <0.001 |
| model 4: VSM | age | 1 (0.02) | [1-1.1] | 0.07 |
|  | FGM | 1.00 (reference group) |  |  |
|  | SGM | 0.9 (0.7) | [0.2-3.9] | 0.9 |
|  | T/E | 2 (0.9) | [0.8-5] | 0.2 |
|  | Strongly positive serology | 1.00 (reference group) |  |  |
|  | Negative serology | 1.2 (0.7) | [0.3-3.9] | 0.8 |
|  | Positive serology | 1.4 (0.8) | [0.5-4.3] | 0.5 |
|  | Pseq | 1 (0.02) | [1.0-1.1] | 0.1 |

FGM, first-generation of migrants; SGM, Second-generation of migrants; T/E, Travellers/expatriates; VSM, very severe malaria; CI, Confidence interval; Ptot, estimated total parasite biomass; Pcirc, estimated total circulating biomass; Pseq, estimated sequestered parasite biomass.

Multivariate analysis was performed on 315 patients for PfHRP2 (model 1) and 176 patients for Ptot, Pcirc and Pseq (model 2-4 respectively). Age, malaria exposure, serology status and log-transformed PfHRP2, Ptot, Pcirc and Pseq were selected, considered as influent factors. FGM and strongly positive serology were chosen as reference group for the analysis whereas age, PfHRP2, Ptot, Pcirc and Pseq were considered as continuous variable. Factors have a significant influence if p value<0.05.
